# Depression and death anxiety among patients undergoing hemodialysis during the COVID-19 pandemic in Palestine: a cross sectional study

**DOI:** 10.3389/fpsyt.2023.1247801

**Published:** 2023-08-31

**Authors:** Mohammed Ibrahim, Elias Saeed, Islam Hamarsheh, Hamzeh Al Zabadi, Muna Ahmead

**Affiliations:** ^1^Faculty of Medicine, Al-Quds University, Jerusalem, Palestine; ^2^Department of Public Health, Faculty of Medicine and Health Sciences, An-Najah National University, Nablus, Palestine; ^3^Faculty of Public Health, Al-Quds University, Jerusalem, Palestine

**Keywords:** COVID-19, pandemic, hemodialysis, depression, death anxiety

## Abstract

**Background:**

Hemodialysis patients are vulnerable to serious complications such as prolonged hospital stay and psychosocial issues like depression and death anxiety. Studies on psychosocial factors on end-stage renal disease patients’ outcomes during COVID-19 pandemic are limited. We aimed to determine the prevalence of depression and death anxiety among Palestinian hemodialysis patients and the evaluate the relationship between their sociodemographic and clinical characteristics during COVID-19 Pandemic.

**Methods:**

A cross-sectional study was conducted using a convenience sampling technique. We recruited 308 hemodialysis patients from five hemodialysis units located in government hospitals in Palestine. Beck Depression Inventory and the Templers Death Anxiety Scale were used to collect data, which were then analyzed using SPSS version 20. Descriptive statistics (frequencies and means), t-test, ANOVA and multiple linear regression models were used for data analysis.

**Results:**

Nearly 66.2% of the sample had depression symptoms, 61.4% met the diagnostic threshold for depression, and 69.8% had death anxiety. Furthermore, the multivariate analysis revealed that having a female identity, residing in a city or refugee camp, and patients who reported not experiencing depression had a significant relationship with death anxiety, while having a higher educational level than 12 years, having one or more chronic co-morbidities, and patients who reported experiencing death anxiety had a significant correlation with depression.

**Conclusion:**

Patients receiving hemodialysis frequently experience depression and death anxiety. These patients should receive a psychiatric evaluation in the early stages of their illness so that timely and appropriate psychological interventions can be given in hemodialysis facilities in Palestine during and after future pandemics.

## 1. Background

COVID-19 was declared a pandemic by the World Health Organization (WHO) in March of 2020 (1). First identified in Wuhan, China, in December of 2019, COVID-19 rapidly spread worldwide with prominent consequences for health and healthcare systems (2). The state of emergency was declared in Palestine on March 5th, 2020. As a result, people were advised to self-quarantine in their homes and not to go out unless absolutely necessary. Since then, the number has risen, with 480,581 laboratory-confirmed COVID-19 cases and 5,042 deaths reported by January 2022 (3). With the fast spread of COVID-19, global health systems face significant challenges in containing infections, detecting and managing COVID-19 patients, and ensuring effective public-health strategies (4, 5). While arising from an infectious illness with predominant physical health implications, these repercussions could have also significantly impacted patients’ mental health and wellbeing, particularly those with chronic kidney disease (CKD) (6–10).

Female gender, younger or older age, prior psychiatric history, physical or mental health issues, economic insecurity, high morbidity and mortality, and exorbitant healthcare costs are all linked to CKD (11–16). Worldwide, the prevalence of CKD had been rising by 8% per year (17), and hemodialysis and kidney replacement therapy are used by almost 4 million people worldwide (18). According to the Palestinian Ministry of Health, there are an increasing number of ESRD patients who require dialysis. In Palestine, there were 1,014 dialysis patients in 2015 and 1,557 in 2020, with an average of 5.3 patients per machine (19, 20).

Patients undergoing dialysis were found to be susceptible to COVID-19, which could have increased their risk of poor prognosis and serious complications such as extended hospital stay, admission to the intensive care unit, and death (21, 22). High burden of symptoms, including depression and anxiety, as well as psychosocial issues were also noted (23). During COVID-19, these patients must have used public transportation to travel to dialysis appointments at least three times per week. Families of patients may be denied access to dialysis units, which add to the difficulty of maintaining social distance and wearing a face mask while undergoing treatment. Patients may have experienced worsening in their health status and/or developing new symptoms of depression, anxiety, and poor sleep as a result of these conditions. Additionally, in an effort to reduce virus exposure, they might skip treatment appointments, which would increase their risk of hospitalization and mortality. Moreover, some patients may have experienced challenging in financial circumstances that influenced their access to food and their living arrangements, and aggravating psychological symptoms (24). One of the most prevalent and serious psychological issues affecting hemodialysis patients is depression which is inked to poor quality of life and death (24, 25). It has been estimated that (20–30) % of patients receiving chronic dialysis suffered from depression (26). A study in Jordan found that 30% of hemodialysis patients had depression (27) and in Iraq, 80% of patients were found to be depressed (28).

Fear of death or death anxiety which is defined as a feeling of dread, anxiety, or fear of the thought of death or any idea pertaining to dying (29), is another psychological issue that dialysis patients may experience. People who are under a lot of stress from their medical condition may be more prone to having thoughts of death, which increases their stress from the illness (30). According to literature, 60.4% of hemodialysis patients experience death anxiety (25). Hemodialysis patients’ anxiety about dying may be brought on by worries about infections, cancer, liver failure, pulmonary embolism, anemia, high serum phosphate levels, malnutrition, gastrointestinal bleeding, and mental anguish (31–33). Furthermore, the fact that chronic renal failure is progressive, irreversible, and in its advanced stages, affects a person’s function and quality of life may be contributing to this high level of depression and death anxiety (34).

Few studies, however, investigated the COVID-19 outbreak’s mental health issues and risk factors in dialysis patients (35). Only three of the five studies (2, 24, 35–37) that examined the relationship between the COVID-19 pandemic and mental health issues in dialysis patients compared post-pandemic findings with pre-pandemic data. In a study of mental health before and after the COVID-19 pandemic, Bonenkamp et al. (2) found no significant differences in HRQoL or symptoms of mental illness like feeling anxious, depressed, or nervous, worrying, and having trouble falling asleep or staying asleep. Similarly, Nadort et al. (35) found no clinically significant differences between the first and second COVID-19 waves and the pre-pandemic period in terms of the severity of the symptoms of depression, anxiety, and HRQoL in hemodialysis patients. According to the study findings, hemodialysis patients had high levels of pre-existing depression, anxiety, and HRQoL prior to the COVID-19 outbreak (35). Similar findings were reported by Uchida et al. (37), who also found no significant differences in the prevalence of depressive symptoms between the COVID-19 pandemic and the period before it.

The effect of psychosocial factors on the outcomes of ESRD patients during the COVID-19 outbreak in Palestine was not well-studied. To the best of our knowledge, no prior study had been done to evaluate depression and fear of death among patients receiving hemodialysis during the COVID-19 outbreak in Palestine. Similar to that, no other studies evaluated those patients before the pandemic in Palestine. In 2015, Al-Jabi et al. (38) conducted a study with 286 patients to evaluate depression and HRQoL among Palestinian hemodialysis patients. The results showed that the prevalence of depression was 73.1% (38). The current study aimed to assess the prevalence of depression and fear of death among Palestinian hemodialysis patients and its correlation with patients’ sociodemographic and clinical characteristics within the COVID-19 pandemic. Healthcare services by professionals could be enhanced by identifying depressed ESRD patients and their fear of dying in order to enhance the healthcare system and treatment results. The results of our study may also be used to develop effective medical and psychological interventions for those subgroups of patients with chronic diseases in the event of future pandemics, thereby reducing the need for hospitalization and even preventing death.

## 2. Materials and methods

### 2.1. Study design, population and settings

A cross sectional was conducted between December 2020 and March 2021, this study was conducted during the COVID-19 pandemic. It included patients with ESRD who were 18 years of age or older and receiving hemodialysis at dialysis centers in five governmental hospitals (Palestine Medical Complex, Jenin Governmental Hospital, Tulkarm Governmental Hospital, Bethlehem Governmental Hospital, and Hebron Governmental Hospital).

### 2.2. Sample size and technique

The total hemodialysis patients in West Bank in 2019 was 1,545 (20), according to a report from the Palestinian Ministry of Health. Nearly, 308 subjects made up the sample size according to the following criteria: 0.05 significance level, 95% confidence level, 50% response distribution, and 0.05 precision error.

The participants were asked to complete the survey if they could read and write and were in adequate health. Patients who were unable to give informed consent or accurately complete the questionnaires such as cognitively impaired individuals were excluded from the study. The medical staff at these facilities assisted the researchers in selecting participants who met the inclusion criteria. The researchers approached 308 participants in hemodialysis units using a convenience sampling approach, and the participants completed the questionnaire by themselves with a response rate of 100%.

### 2.3. Data collection tools

The data was collected using a questionnaire consisted of three sections with a total question number of 42. The first section included socio-demographic factors (age, gender, city, marital status, home companions, education level, residency, and occupation) as well as medical history (period on dialysis, comorbidities, SARS-CoV-2, quarantine time, the experience of psychological symptoms during quarantine, and seeking psychological intervention).

The second section included the Templer Death Anxiety Scale (Templer DAS) which is based on a two-factor model of death anxiety that includes psychological (internal) and life experience (external) factors related to death (39). It had 15 questions assessing absolute death anxiety, fear of patience and pain, death-related thoughts, time passing and short life, and the fear of the future. Each item had two possible answers (yes, no), which were given the value of 1 and 0, respectively and the true response indicating the presence of anxiety in the participant.

The minimum and maximum possible scores for the Templer DAS are 0 (absence of death anxiety) and 15 (highest level of death anxiety) with cutoff score of 6 such that the scores above and under 6 represent high and low levels of death anxiety, respectively. The presence of death anxiety class interval ranges as follows: from (0–6) refers to the absence of death anxiety, from (7–8) indicates that there is an average concern about death, and from (9–15) indicates the presence of deep concern to death.

The third section had the Beck Depression Inventory (BDI-II) which was developed by Aaron T. Beck in 1961 (40). BDI-II has 21-items that were modified later to measure the intensity and severity of depression symptoms related to emotional, cognitive, and physical symptoms experienced by the participant during the previous 2 weeks. They include sadness, pessimism, sense of failure, loss of pleasure, guilt, an expectation of punishment, dislike of self, self-accusation, suicidal ideation, episodes of crying, irritability, social withdrawal, indecisiveness, worthlessness, loss of energy, insomnia, irritability, loss of appetite, preoccupation, fatigue, and loss of interest in sex (41). Each item is scored from 0 to 3 with a minimum total score of 0 and a maximum score of 63. The total score of 0–13 is considered a minimal range, 14–19 is mild, 20–28 is moderate, and 29–63 is severe (40).

A committee of three mental health experts reviewed the scale’s contents because it had not been previously tested in the Palestinian culture to make sure that the tool is culturally appropriate and no changes were done. The scale was first translated into Arabic by the research team, and then it was reverse translated to English by a licensed medical translator. At the pilot stage, we administered the tool with10 patients to test for language clarity, both the original English questionnaire and the back translated version were examined to ensure that the translation was accurate. The Cronbach’s Alpha reliability test was 0.79 and 0.80 for the Templer Death Anxiety Scale and the Beck Depression Inventory, respectively, indicating good reliability.

### 2.4. Data analysis

Data analysis was performed using the Statistical Package for Social Sciences (SPSS) version 20. Descriptive statistics (frequencies and means) were calculated to assess the demographics and socioeconomic factors. The associations between socioeconomic factors and medical history with the Beck Depression Inventory and Death Anxiety Scale were assessed using t-test and one-way analysis of variance (ANOVA). To determine the predictors of both continuous scale scores of death anxiety and depression, we developed multiple linear regression models including all variables found to be significant in the bivariate analysis with *p*-value less than 0.05.

## 3. Results

According to the analysis of the baseline data, 55.5% of respondents were male, 72.7% had received dialysis for more than a year, 65.6% were married, and 60.4% were from villages. In addition, 80.2% reported that they had no coronavirus infection, 42.5% reported they had previous depression history, 29.9% indicated they had previous death anxiety history, and only 7.5% were found to have sought counseling, as shown in the Table 1. The mean ± standard deviation of death anxiety score and depression score were 7.45 ± 2.52 and 20.1 ± 11.4; respectively (Table 1).

**Table 1:** Simple linear regression for socio-demographic and history factors association with death anxiety and depression scores.

Variable	*N* (%)	Death anxiety scores				Depression scores			
		Mean ± SD	B ± SE	Beta	*p* value (95% CI)	Mean ± SD	B ± SE	Beta	*p*-value (95% CI)
Age									
18 years <50 years^a^	164 (53.2)	7.4 ± 2.7	–	–	–	19.8 ± 11.6	–	–	–
50 years or more	144 (46.8)	7.4 ± 2.3	−0.02 ± 0.3	−0.004	0.95 (−0.6–0.5)	20.4 ± 11.2	0.65 ± 1.3	0.028	0.6 (−1.9–3.2)
Gender									
Male^a^	171 (55.5)	7. ± 2.7	–	–	–	20.4 ± 11.1			
Female	137 (44.5)	8 ± 2.2	0.9 ± 0.3	0.2	0.001 (0.4–1.5)	19.8 ± 11.7	−0.06 ± 1.3	−0.03	0.65 (−3.2–2)
City of living									
Ramallah	81 (26.3)	7.4 ± 2.4	–	–	0.614	18.8 ± 10.8	–	–	0.655
Jenin	12 (3.9)	8.3 ± 2.8	–	–		19.8 ± 12.1	–	–	
Tulkarm	43 (14)	7.5 ± 2	–	–		20. ± 10.2	–	–	
Bethlehem	75 (24.4)	7.3 ± 2.8	–	–		19.9 ± 11	–	–	
Hebron	93 (30.2)	7.4 ± 2.6	–	–		21.6 ± 12.8	–	–	
Quarantine duration									
Less than one month^a^	106 (34.4)	7.4 ± 2.7	–	–	–	20.4 ± 10.8	–	–	–
One month or higher	202 (65.6)	7.5 ± 2.4	0.1 ± 0.3	0.023	0.7 (−0.5–0.7)	19.9 ± 11.7	−0.48 ± 1.4	−0.02	0.7 (−3.2–2.2)
How long have you been on Dialysis?									
Less than 1 year^a^	84 (27.3)	7.5 ± 2.4	–	–	–	19.1 ± 11	–	–	–
One year or more	224 (72.7)	7.4 ± 2.6	−0.02 ± 0.3	−0.004	0.945 (−0.7–0.6)	20.5 ± 11.5	1.4 ± 1.5	0.054	0.3 (−1.5–4.3)
Marital status									
Married^a^	202 (65.6)	7.5 ± 2.7	–	–	–	20.4 ± 10.8	–	–	–
Un-married	106 (34.4)	7.4 ± 2.2	−0.08 ± 0.3	−0.015	0.8 (−0.7–0.5)	19.5 ± 12.5	−0.9 ± 1.4	−0.37	0.5 (−3.6–1.8)
Do you live alone?									
Yes^a^	49 (15.9)	7.3 ± 2.7	–	–	–	22.1 ± 11.8	–	–	–
No	259 (84.1)	7.5 ± 2.5	0.14 ± 0.4	0.021	0.7 (−0.6–0.9)	19.7 ± 11.3	−2.4 ± 1.8	−0.076	0.2 (−5.9–1.1)
Residency									
Village^a^	186 (60.4)	7.1 ± 2.5	–	–	–	20.4 ± 11.5	–	–	–
City and/or refugee camp	122 (39.6)	8 ± 2.4	−0.47 ± 0.3	−0.103	0.1 (−1–0.04)	19.6 ± 11.3	−0.8 ± 1.3	−0.036	0.5 (−3.5–1.8)
Educational level									
1–12 years (School education)^a^	220 (71.4)	7.4 ± 2.7	–	–	–	21.1 ± 11.9	–	–	–
> 12 years of education (University education)	88 (28.6)	7.5 ± 2.1	0.06 ± 0.3	0.01	0.9 (−0.6–0.7)	17.5 ± 9.6	−3.7 ± 1.4	−0.145	0.01 (−6.5– −0.9)
Work status									
Employed^a^	72 (23.4)	7.5 ± 2.1	–	–	–	18.3 ± 10.4	–	–	–
Unemployed	236 (76.6)	7.4 ± 2.6	−0.104 ± 0.3	−0.02	0.8 (−0.8–0.6)	20.6 ± 11.7	2.3 ± 1.5	0.085	0.13 (−0.7–5.3)
No. of chronic diseases that patients suffer from									
No previous chronic co-morbidities^a^	52 (16.9)	7.0 ± 3.1	–	–	–	15.9 ± 10.8	–	–	–
One or more chronic co-morbidities	256 (83.1)	7.5 ± 2.4	0.516 ± 0.4	0.077	0.2 (−0.2–1.3)	21 ± 11.3	5.1 ± 1.7	0.168	0.003 (1.7–8.5)
Have you ever had the Corona virus infection?									
Yes^a^	61 (19.8)	7.7 ± 2.1	–	–	–	17.9 ± 12.4	–	–	–
No	247 (80.2)	7.4 ± 2.6	−0.279 ± 0.4	−0.044	0.4 (−1–0.4)	20.6 ± 11	2.8 ± 1.6	0.098	0.06 (−0.4–6)
Have you suffered from depression in the past?									
Yes^a^	131 (42.5)	7.8 ± 2.6	–	–	–	24.1 ± 11.7	–	–	–
No	177 (57.5)	7.2 ± 2.4	−0.615 ± 0.3	−0.121	0.03 (−1.2– −0.05)	17.2 ± 10.2	−6.9 ± 1.3	−0.301	0.00 (−3.4– −4.5)
Have you suffered from death anxiety in the past?									
Yes^a^	92 (29.9)	8.2 ± 2.1	–	–	–	25.3 ± 11.9	–	–	–
No	216 (70.1)	7.1 ± 2.6	−1.08 ± 0.3	−0.197	0.001 (−1.7– −0.5)	17.9 ± 10.5	−7.5 ± 1.4	−0.3	0.00 (−10.1– −4.8)
Have you received psychological help?									
Yes^a^	23 (7.5)	7.8 ± 2.1	–	–	–	20.3 ± 13.1	–	–	–
No	285 (92.5)	7.4 ± 2.5	−0.409 ± 0.5	−0.043	0.5 (−1.5–0.7)	20.1 ± 11.3	−0.2 ± 2.5	−0.004	0.9 (−5.1–4.7)

a Reference category; *p* value less than 0.05.

Additionally, simple linear regression revealed that there were significant relationships between having a high level of education (more than 12 years), having one or more chronic co-morbidities, experiencing depression, and having death anxiety. Additionally, a significant relationship between depression and gender (females) and death anxiety were found as seen in Table 1.

**Figure 1:**
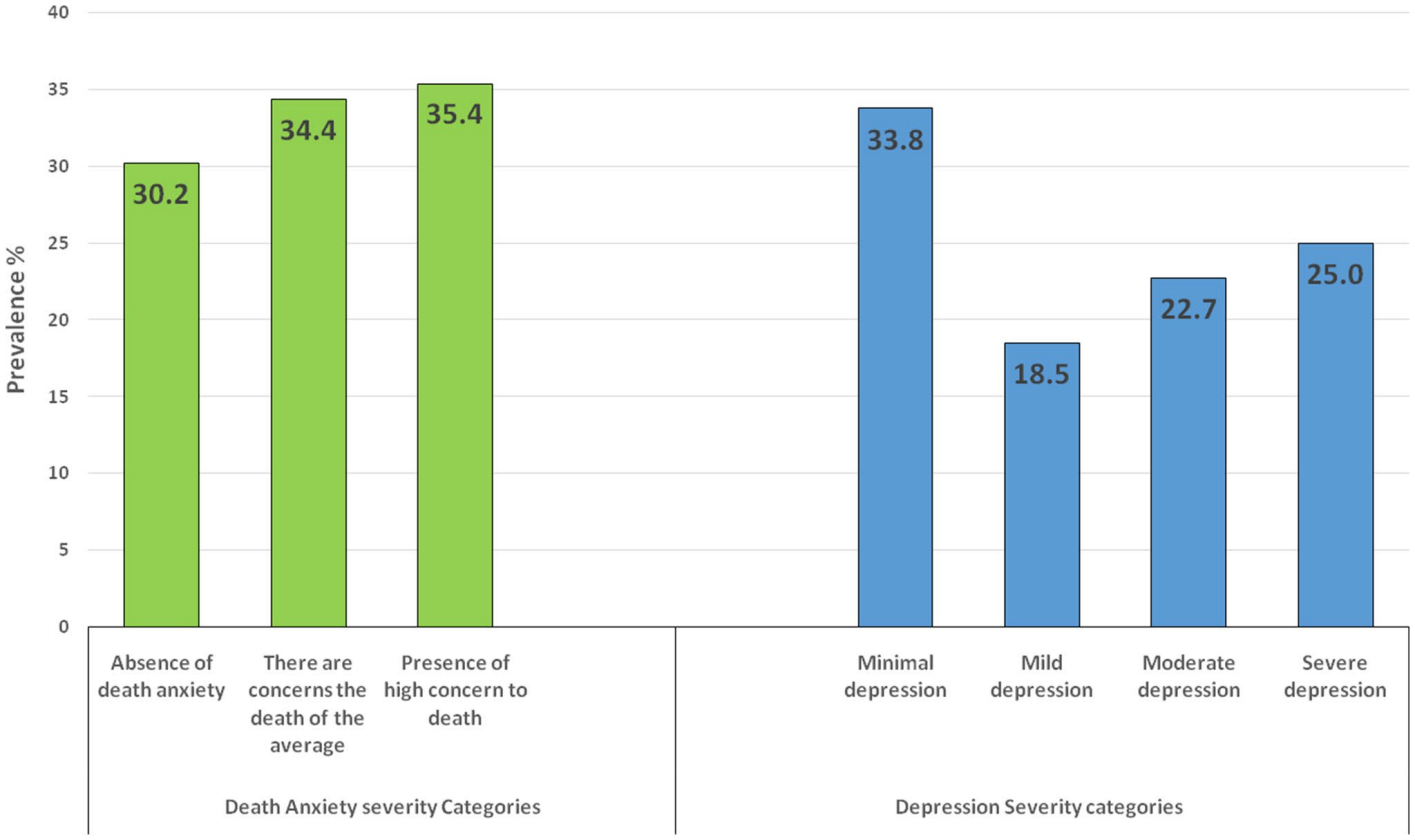
Prevalence of death anxiety and depression score categories.

Furthermore, Figure 1 shows that 30.2% of the participants had an absence of death anxiety, 35.4% had a high concern of death anxiety, and 34.4% had average death anxiety. This indicated that 69.8% of the sample had death anxiety.

Regarding depression, 25% had severe depression symptoms, while 33.8% had only mild depression symptoms. In general, the results indicated that 66.2% of the sample exhibited symptoms of depression.

Moreover, the multivariate analysis showed that being a female, living in a city or a refugee camp, and not suffering from depression had a significant relationship with death anxiety, while an educational level of more than 12 years, suffering from one or more chronic co-morbidities, and suffering from death anxiety had a significant relationship with depression as shown in Table 2.

**Table 2:** Multivariate linear regression analysis for factor^a^ associated with death anxiety and depression scores.

Variable	B	SE	Beta	95% CI	*p*-value
Death anxiety model					
Gender (Female)	0.92	0.28	0.18	0.37–1.47	0.001
Residency (City or Ref. Camp)	0.89	0.28	0.17	0.33–1.45	0.002
Depression (No)	−0.62	0.28	−0.12	−1.17 – −0.07	0.028
Constant	5.85	0.73	–	4.4–7.3	<0.001
Depression model					
Educational level (>12 years)	−3.2	1.35	−0.13	−5.8 – −0.523	0.019
Co-morbidities (One or more)	4.4	1.3	0.15	1.2–7.6	0.007
Death anxiety (No)	−7.2	1.3	−0.29	−9.8–−4.6	<0.001
Constant	28.26	4.3	–	19.82–36.70	<0.001

a Reference categories: Gender: Male; Residency: Village; Depression: Yes; educational level: <1–12 years (School education); Co-morbidities: No previous chronic co-morbidities’ Death anxiety: Yes.

**Figure 2:**
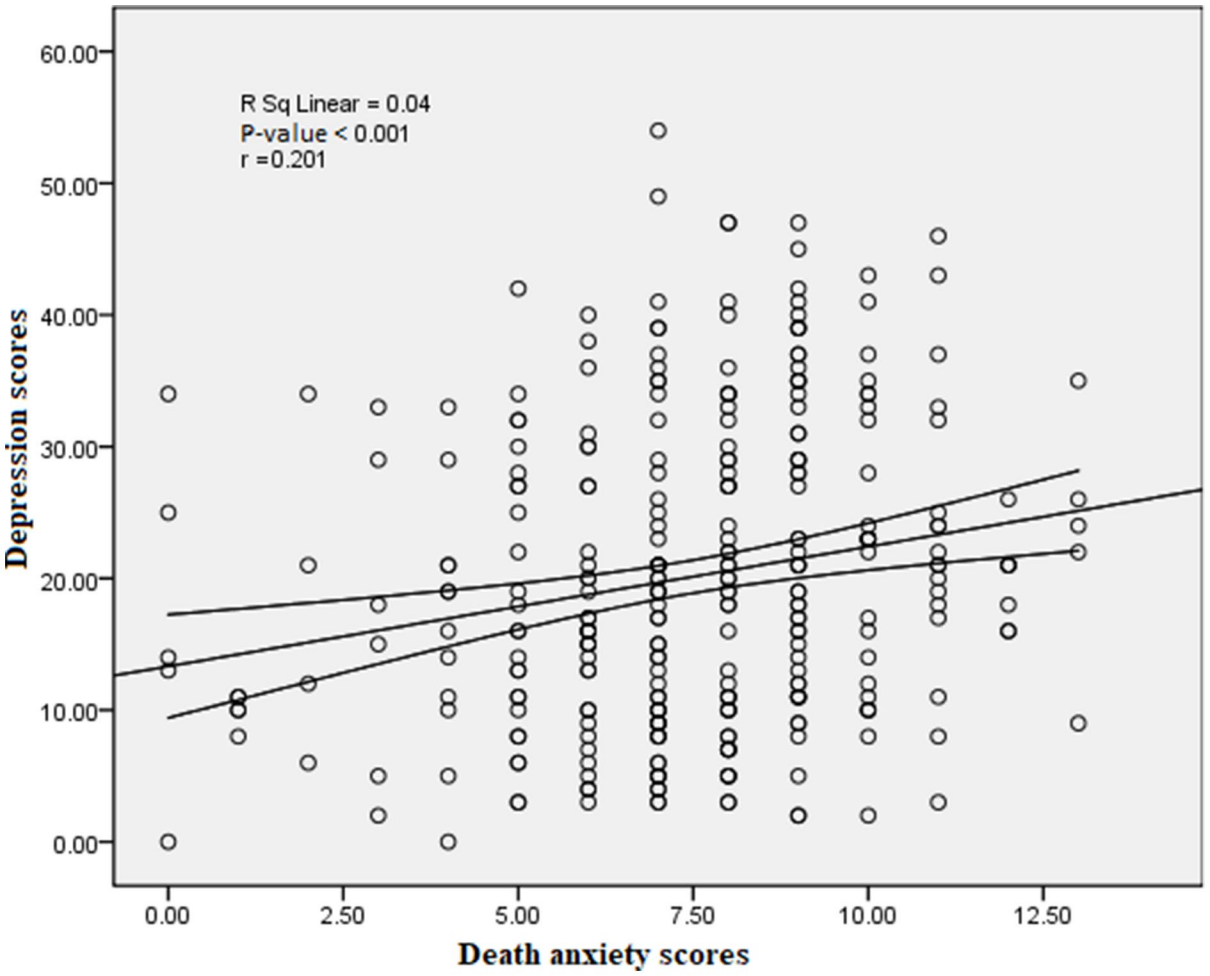
Scatter-plot correlation between death anxiety and depression scores. Lines represent 95% CI of the mean difference.

For the relationship between depression and death anxiety, scatterplot correlation showed a significant positive relation (r = 0.201) (*p*-value<0.001) as seen in Figure 2.

## 4. Discussion

This study aimed to determine whether patients receiving hemodialysis during COVID-19 pandemic experienced a high prevalence of depressive symptoms and death anxiety. Studies found a rise in mental health issues compared to the pre-pandemic period (8–10), especially depression and anxiety in hemodialysis patients, which led to more hospitalization and an increased risk of death (24, 42–44). There is a lack of studies that assessed death anxiety in hemodialysis patients during COVID-19 pandemic particularly in Palestine. Our study was the first to assess death anxiety prevalence rates and its relation with depression in hemodialysis patients in Palestine during COVID-19 pandemic.

The current study found that during COVID-19 pandemic, hemodialysis patients in Palestine had high levels of death anxiety and depressive symptoms as 69.8% of the sample reported death anxiety, and 66.2% reported depressive symptoms. Similarly, Ghiasi et al. (25) found that 60.4% of the patients had high levels of death anxiety in Iran and in Lebanon, Khoury et al. (45) reported a high rate of depression in patients on hemodialysis (57.1%). In addition, Duru (46) revealed that rates of depression were significantly higher both before and after COVID-19 pandemic in Turkey (63.1% vs. 75.0% as overall, and 19.0% vs. 33.3% for moderate-to-severe depression). The Beck Depression Inventory scale and repeated exposure to traumatic war-zone conflicts or events have been suggested as possible explanations for the higher rates in these studies (45). Therefore, it is critical to assess depression and death anxiety in these patients because they have been linked to poor survival rates, a high risk of suicidal ideation, and non-adherence to therapy (47–49).

In contrast, the prevalence of depressive symptoms during COVID-19 pandemic among Japanese hemodialysis patients was lower (26.1%) than that of the current study participants according to Uchida et al. (37). In China, Hao et al. (50) found that among Chinese patients, anxiety or depressive symptoms were reported by 34.89 and 30.02% of patients; respectively. Additionally, Meng et al. (51) revealed that depression was prevalent in 55.1% of cases, with mild, moderate, and severe disorders accounting for 27, 5, 21, and 6.6% of those cases; respectively. Moreover, the results of the present study were higher than those of studies from other Arab countries. For example, according to Al-Shammari et al. (52), hemodialysis patients in Kuwait had prevalence rates of depression and anxiety during COVID-19 outbreak of 21.7 and 21.4%; respectively (51). In Saudi Arabia’s Jazan region, patients receiving hemodialysis had a depression prevalence of 43.6%, with 12.8% of them reported mild depression, 15.6% reported moderate depression, and 15.1% reported severe depression (52). In Oman, Al Naamani et al. (53) study, reported that 43.9% of patients undergoing hemodialysis had anxiety and 33.3% had depression. Even before COVID-19 pandemic, some studies from other Arab countries revealed a low prevalence of anxiety and depression. For example, El Filali et al. (54) in Morocco found that major depressive episode (MDE) prevalence was 34%, and anxiety disorder prevalence was 25.2%, while a Turkistani et al. (55) in Saudi Arabia found that 21.1% of patients had anxiety and 23.3% had depression.

Studies reported that this low level of depression prevalence might be due to that hemodialysis patients were less likely to have their daily routines disrupted by travel restrictions and the nationwide lockdown and because of the high levels of pre-pandemic depression and anxiety (2, 35). Other researchers contend that these patients may be resilient, able to handle stress by developing and using coping mechanisms that help them deal with various stressors like COVID-19 pandemic (2, 56).

According to the our study, a high percentage of Palestinian hemodialysis patients had depressive symptoms, which was higher than the global prevalence for the general population (3.7–48.3%) (57) and was consistent with findings from Al-Jabi et al. (38), who claimed that prior to COVID-19 pandemic, 73.1% of Palestinian hemodialysis patients had depression. Despite that the causes of the high levels of depressive symptoms and death anxiety during COVID-19 pandemic were not investigated in our study, other research in the dialysis population suggested a number of factors. For instance, COVID-19 pandemic made it more difficult for Palestinian hemodialysis patients to access care because of the country’s underdeveloped healthcare system and difficult political conditions. Also, COVID-19 crisis had revealed significant flaws in Palestine’s social and public health systems, including social exclusion, inequality, fragility, lack of preparation, underinvestment, and a severe lack of COVID-19 tests, sanitation, hygiene products, ventilators, and ICU beds (58).

Additionally, the Palestinian Authority placed Palestine under an internal lockdown, and Israel imposed an external closure, both of which had a detrimental impact on the nation’s economy and social life. Moreover, the persistent rise in poverty and unemployment as well as the prolonged cuts in foreign aid for healthcare services made the situation in Palestine worse both during and even before COVID-19 pandemic (59, 60). Therefore, the complicated political, social, and economic conditions that existed prior to COVID-19 pandemic, and the quarantine itself, had a significant negative impacts on the post COVID-19 mental health of patients with renal diseases (61).

Another significant factor that may contribute to a high level of depression and death anxiety among patients receiving hemodialysis could be the rising in death rate and infection during COVID-19 pandemic (62–64). Because of their use of public transportation and inability to maintain social distance from one another in treatment units during the pandemic, this could have causes a significant risk of COVID-19 transmission (65–67). The lack of knowledge regarding the disease’s transmission and treatments at the beginning of the pandemic could also have contributed to an increase in people’s depressive symptoms and death anxiety (68). According to Lee et al. (24), more than 85% of patients, particularly those who were infected with the disease, expressed anxiety regarding going to dialysis sessions (24, 69).

Interestingly, despite having high levels of depression and death anxiety, only a small proportion of hemodialysis patients seeked psychological counseling. For example, only 7.5% of patients in this study reported seeking psychological assistance or treatment. In contrast, Lee et al. (24) found that 98% of the participants attended telemedicine consultations with mental or health care professionals from home (71% video visits and 27% phone visits). It had been found that hemodialysis patients who have more social support were less likely to experience depression and anxiety (26). Greater psychological adjustment, an improvement in the patients’ ability to handle routine care, and an increase in treatment compliance were all correlated with improved social support (62, 70). Given that 76.6% of the participants in the current study reported being unemployed, their living situation and nutritional status may be impacted. In hemodialysis patients, nutritional status had also been connected to anxiety and depression. A sample of 55 adult hemodialysis patients revealed that those with poor nutritional status had significantly higher prevalence rates of depression and anxiety (71). Therefore, these results may indicate the need to improve the financial situation of these patients as well as provide them with social and psychological support.

In addition, the results of the multivariate analysis indicated that there was a strong relationship between being a woman and fear of death. Ghiasi et al. (25) found that having a higher income and being a man were related to death anxiety. In light of the female gender role’s association with emotional behavior and the likelihood that women will express their anxiety about dying more readily than men, according to Dönmez et al. (72), this gender difference may be due to cultural considerations.

The multivariate analysis also revealed a significant correlation between living in a city or a refugee camp and fear of dying. One possible explanation could be that people’s businesses and income were negatively impacted by the closure of cities and refugee camps, and their anxiety about death and food insecurity increased. Also, living in overcrowded conditions, especially in refugee camps, increases the risk of COVID-19 transmission and infection, which might be contributed to an increased level of death anxiety. The socioeconomic status of patients and their degree of death anxiety was not, however, significantly correlated, according to Karaca et al. (73). However, Al-Jabi et al. (38), reported a relationship between depression and living in rural areas/camps in Palestine.

Moreover, in our study, there was a significant correlation between depression and having more than 12 years of education. In contrast, lower educational status was found to be linked to higher depression scores by Dönmez et al. (72) and Nelson et al. (74). Al-Jabi et al. (38) found no correlation between education level and depression, but they did find a strong correlation between having one or more chronic comorbidities and depression. On the other hand, Othayq and Aqeeli (75) reported that there was no association between having a high level of education and depression, but that there was a significant link between depression and patients with less education.

Finally, the result of the current study showed a significant relationship between depression and death anxiety. According to earlier research, having more anxiety and depression symptoms was correlated with having more death anxiety (76–78). Additionally, death anxiety has trans-diagnostic components that are crucial for the emergence and severity of depressive symptoms (79). Death anxiety was shown by Menzies et al. (80) to strongly predict psychopathology, such as stress, anxiety, and depression. Ghiasi et al. (25) also reported that the majority of hemodialysis patients displayed signs of death anxiety in addition to having low to moderate quality of life, with the worst reductions occurring in both the psychological and physical domains. It is worth mentioning that, as a trans-diagnostic construct, death anxiety may in many disorders suggest unresolved emotional and physical distress. In addition, according to Iverach et al. (79) and Menzies et al. (80), death anxiety can raise or maintain the risk of developing a number of mental disorders, including anxiety disorders such as generalized anxiety disorder, panic disorders (81–83), depression (84), obsessive–compulsive disorder (85), and posttraumatic stress disorder (86). The morbidity and mortality rates associated with mental illness will decline with early detection and treatment. This may indicate the importance of screening hemodialysis patients for death anxiety in clinical settings to further prevent and limit the emergence of other mental disorders such as GAD, and substance abuse and to improve their adherence to their treatment plan. The findings also may indicate the demand for psychological interventions that target death anxiety in general specifically in hemodialysis patients.

In conclusion, the current study findings highlighted the direct danger that death anxiety poses to mental health, as well as the frequent depression that hemodialysis patients experienced, which might have an impact on their treatment and their quality of life. Patients who are depressed also may have suicidal thoughts and negative attitudes toward death. Therefore, we should be alert for death anxiety and depressed dialysis patients and handle them with extreme caution (87, 88).

This study had some limitations. Making causal inferences is hindered by convenience sampling and cross-sectional designs. As a result, it is important to interpret the study results carefully. Based on the findings, it is difficult to compare this study with other studies because there aren’t many studies that evaluate the fear of dying among hemodialysis patients, particularly during COVID-19 pandemic in Palestine. In addition, self-reported questionnaires rather than psychiatric interviews were used to assess depression and death anxiety. Furthermore, it is challenging to pinpoint the precise effects of the pandemic on the mental health of Palestinian hemodialysis patients due to the scarcity of studies among them prior to and during COVID-19 pandemic. The study also did not take other mental illnesses into account, most notably generalized anxiety disorder, which could have contributed to the development of COVID-19 related death anxiety. The current study also did not examine the factors, such as the economic or political environment, that might account for the high levels of death anxiety and depression among Palestinian hemodialysis patients.

Nevertheless, despite these limitations, our research on the effects of the COVID-19 pandemic on the mental health of hemodialysis patients (depression and death anxiety) still makes a significant contribution to the literature.

## 5. Implications of the study

Patients receiving dialysis treatment are at risk of poor prognosis and severe consequences such as prolonged hospitalization, critical care unit admission, and death due to COVID-19 disease. Moreover, many patients have psychosocial issues and a high burden of symptoms such as depression and anxiety. Therefore, it is vital to provide psychological support and interventions to decrease their stress throughout the pandemic. One of the utmost priorities for healthcare institutions, policymakers, and managers is to prepare for the possibility of another epidemic/pandemic in Palestine. Therefore, improving the clinical, social, psychological, and political environment is necessary. Additionally, focusing on those with a high risk for depression and death anxiety, such as patients with high education, living in refugee camps, having comorbidities, and being female is important to face future challenges of pandemics similar to COVID-19.

Future studies should explore death anxiety and its relation with other psychiatric comorbidities in CKD patients, other socio-demographic and medical factors, and their effect on disease progression. Also, further qualitative studies are needed to explore the causes of death anxiety and depression among hemodialysis patients during the pandemic in Palestine. In addition, future research is needed to investigate the effectiveness of different psychological interventions that aim to decrease depression and death anxiety among hemodialysis patients during stressful conditions similar to the COVID-19 pandemic.

## 6. Conclusion

Our study concluded that depression and fear of death were highly prevalent in CKD patients undergoing hemodialysis during the COVID-19 pandemic. Therefore, these patients should undergo a psychiatric evaluation in the early phase of the illness so that timely and appropriate interventions can be conducted, and their quality of life can be enhanced by reducing the psychiatric disorder burden. Also, the study indicates the need for proper psychiatric and psychological treatment in hemodialysis centers in Palestine to treat death anxiety and depression.

## Data availability statement

The original contributions presented in the study are included in the article/supplementary material, further inquiries can be directed to the corresponding author.

## Ethics statement

The studies involving humans were approved by the Institutional Review Board (IRB) at Al-Quds University (November 29, 2020; 158/REC/2020). The studies were conducted in accordance with the local legislation and institutional requirements. The participants provided their written informed consent to participate in this study.

## Author contributions

MI, ES, IH, HA, and MA shared study design. Data gathering was done by MI, IH, and ES. Data analysis was conducted by HA. Initial manuscript draft was done by MA. MI, IH, and ES made minor contributions to initial draft. All authors contributed to the article and approved the submitted version.

## Conflict of interest

The authors declare that the research was conducted in the absence of any commercial or financial relationships that could be construed as a potential conflict of interest.

## Publisher’s note

All claims expressed in this article are solely those of the authors and do not necessarily represent those of their affiliated organizations, or those of the publisher, the editors and the reviewers. Any product that may be evaluated in this article, or claim that may be made by its manufacturer, is not guaranteed or endorsed by the publisher.

## Abbreviations

CKD: Chronic Kidney Disease
ESRD: End-Stage Renal Disease
BDI: Beck Depression Inventory
WHO: World Health Organization
